# Clinical nurses’ beliefs, knowledge, organizational readiness and level of implementation of evidence-based practice: The first step to creating an evidence-based practice culture

**DOI:** 10.1371/journal.pone.0226742

**Published:** 2019-12-26

**Authors:** Jae Yong Yoo, Jin Hee Kim, Jin Sun Kim, Hyun Lye Kim, Jung Suk Ki

**Affiliations:** 1 Department of Nursing, College of Medicine, Chosun University, Gwangju, South Korea; 2 Department of Nursing, Chosun University Hospital, Gwangju, South Korea; University of Adelaide, AUSTRALIA

## Abstract

**Background:**

This study aimed to identify clinical nurses’ evidence-based practice (EBP) knowledge, beliefs, organizational readiness, and EBP implementation levels, and to determine the factors that affect EBP implementation in order to successfully establish EBP. This study was conducted at a university-affiliated tertiary hospital located in a provincial area in Korea. The research design was based on Melnyk and Fineout-Overholt’s Advancing Research & Clinical Practice through Close Collaboration model as the first step.

**Methods:**

A descriptive and cross-sectional design was conducted and a convenience sample of 521 full-time registered nurses from an 849-bed tertiary hospital were included. Structured questionnaires were used to assess EBP knowledge, EBP beliefs, organizational culture & readiness and EBP implementation. Data were analyzed using SPSS V 25.0 by using descriptive and inferential statistics and hierarchical multiple regression was performed to determine the factors affecting the implementation of EBP.

**Results:**

Our findings showed that the clinical nurses had a positive level of EBP beliefs, but the level of EBP knowledge, organizational readiness and EBP implementation were insufficient. EBP knowledge, beliefs, and organizational readiness were significantly positively correlated with EBP implementation. In the final model, EBP knowledge and organizational readiness were significant predictors of EBP implementation; the model predicted 22.2% of the variance in implementation.

**Conclusions:**

Based on these results, the main focus of the study was the importance of individual nurses' efforts in carrying out EBP, but above all efforts to create an organizational culture to prepare and support EBP at the nursing organization level. In the initial process of introducing and establishing EBP, nurse administrators will need to minimize expected barriers, enhance facilitators, and strive to build an infrastructure based on vision, policy-making, budgeting, excellent personnel and facilities within the organization.

## Introduction

Evidence-based practice (EBP) is a problem-solving approach to clinical care that incorporates the conscious use of the best available scientific evidence, clinicians’ expertise, and patients’ values [1]. This leads to safe patient care and positive patient outcomes, reduces nursing time and medical costs through standardization of nursing practice [2–5]. It also improves professional autonomy and job satisfaction for clinical nurses, ultimately bringing potential benefits to patients, nurses and the health care system [6–8]. For this reason, EBP has emerged as a central concept in the planning and implementation of healthcare systems worldwide. As EBP rapidly replaces the traditional paradigm of authority in healthcare decision-making, health professionals have an obligation to access knowledge, apply it in practice, and lead others to use it appropriately [8–10].

Western countries, such as the United States (US), United Kingdom (UK), and Australia, have emphasized nursing through EBP since the 1990s, and there are active movements such as developing evidence-based guidelines providing various resources related to EBP from organizations specialized in EBP (Cochrane, Joanna Briggs Institute, etc.) [11, 12]. In addition, the Institute of Medicine presented EBP competency as one of the five core competencies of healthcare professionals [13], and the American Association of Colleges of Nursing also presented EBP as one of the nine essential elements of professional nursing practice [14]. Over the past 30 years, there has been marked theoretical and practical growth associated with EBP, including education and training for EBP in nursing practice, and research conducted including various facilitation strategies [9, 15].

In Korea, however, EBP in nursing was first introduced in the early 2000s [16]. Awareness on the importance of EBP has spread around major large tertiary hospitals in Seoul, but the actual performance of EBP has been reported to be poor outside of the metropolitan area [17–19]. A study involving 437 nurses at tertiary hospitals conducted in 2004, which was the very first time the concept of EBP was introduced in Korea, found that 58% of nurses did not perform nursing practice according to the latest guidelines [20]. Korean nurses were reported to be underperforming EBP until recently [19]. In 2013, only 12 of the 30 tertiary hospitals surveyed (40.0%) were organized by EBP committees and were conducting EBP-related clinical nursing studies [18]. Although various efforts have been made to promote EBP in Korea in recent years, it is apparent that institutional support for EBP is not systematic and insufficient throughout the country.

Barriers to conducting EBP for Korean nurses include: the lack of knowledge and skills, lack of belief and capacity, lack of database access and utilization, and insufficient critical thinking and motivation [20–22]. The barriers to conducting EBP in Korea at the organizational level are organizational culture, insufficient education programs, lack of well-trained EBP experts, lack of time, and inadequate communication [20, 21, 23]. While EBP is a valuable concept, it is difficult for a nurse to implement it first before a nursing organization embraces this new concept [24]. Therefore, for a successful implementation of EBP, the readiness of an individual nurse and organization must be assessed.

First, EBP implementation is influenced by the knowledge, skills, and beliefs of the individual nurse on EBP [22, 25, 26]. At the organizational level, it is necessary to create an organizational culture that strengthens and supports the nurse’s values and beliefs on EBP, and to share the common beliefs or values of its members to achieve the common goal of successful implementation of EBP [12, 27]. It is also important to provide training programs for nurses to strengthen their EBP capabilities and to foster leaders who can effectively lead EBP implementation [7, 28, 29]. There are various strategic models for successful EBP implementation [15]. The Advancing Research and Clinical Practice through Close Collaboration (ARCC^©^) model proposed by Melnyk and Fineout-Overholt [24] is a representative strategic model that emphasizes personal and organizational elements. The ARCC^©^, a strategic model developed by the EBP center of the University of Arizona in the US, proposes the use of methodological strategies to promote the implementation of EBP based on the close cooperation between clinical nurses and researchers [24]. The first step in the ARCC^©^ model is to assess the organizational culture and readiness of the medical institution to successfully establish EBP. This will help identify the strengths and barriers of the organization and improve the nurses’ knowledge on, belief regarding, and capacity to adopt and implement EBP through education and training, environmental improvement, and organizational support while focusing on mentors who act as facilitators in the performance of EBP [24, 30]. Successful implementation of EBP can increase the job satisfaction of professional nurses and ultimately improve nursing-sensitive outcomes [6, 7, 31, 32]. The conceptual framework in this study was constructed based on the ARCC^©^ model.

To date, only a few studies have evaluated the level of preparation, correlation, and influencing factors of EBP implementation among individual nurses and organizations in Korea. This study was conducted at a university-affiliated tertiary hospital located in a provincial area in Korea, as the first step in implementing EBP in accordance with the ARCC^©^ model. This study aimed to identify the clinical nurses’ EBP knowledge, beliefs, organizational readiness, and EBP implementation levels, and to determine the factors that affect EBP implementation in order to successfully establish EBP. The specific objectives of this study were as follows:

1. To identify the clinical nurses’ EBP knowledge, beliefs, organizational readiness, and EBP implementation levels
2. To examine the differences in clinical nurses’ EBP knowledge, beliefs, organizational readiness, and EBP implementation levels based on the general and research-related characteristics of participants and explore the relationships among these variables
3. To identify the factors that affect EBP implementation

## Methods

### Study design and participants

This was a cross-sectional, descriptive study. The participants were recruited from an 849-bed acute care tertiary hospital in South Korea. Convenience sampling was used to select full-time registered nurses employed at this hospital. The sample size required for the multiple regression analysis was calculated using G-Power 3.1 [33], with an effect size of 0.02, significance level of 0.05, and test power of 0.80 with 14 predictors. It was determined that at least 485 participants were required for analysis. However, this study was the first step involved in the ARCC^©^ model, and all nurses were surveyed to identify the current state of nurses belonging to the abovementioned hospital. Among the 632 registered nurses, 82 of the following nurses were excluded from the survey: 1) part-time nurses, 2) nurses participating in training for new nurses without full-time assignments in the hospital, and 3) laboratory and research nurses not involved with direct patient care. A total of 550 questionnaires were distributed; 521 were returned (94.7% response rate). Finally, 521 who fully understood the purpose of this study and voluntarily consented to participate were included. Participants included clinical nurses working in the wards and special units, clinical nurse specialists, nurse managers, and nurse administrators.

### Measurements

This study used structured questionnaires, consisting of the following items: general and research-related characteristics (13), EBP knowledge (14), EBP beliefs (16), organizational readiness for EBP (25), and EBP implementation (18). The measurements used in this study were approved by the original authors and translated versions into Korean have already been used in the previous studies [19, 22, 34]. However, researchers have modified and supplemented some of the items with words or expressions that are commonly used by nurses in this hospital where the study was conducted. Prior to the survey, a pilot test of five clinical nurses identified and revised problematic questionnaire items. The details of the measurements for each variable are as follows.

#### EBP knowledge

Participants’ knowledge in implementing EBP was measured using knowledge-related questions from the Evidence-based Practice Questionnaire, developed by Upton & Upton [35]. This tool consists of 14 items, including “converting your information needs into a research question” and “ability to analyze critically, evidence against set standards.” Response scores on the scale range from 1 (very lacking) to 7 (excellent). Possible total scores range from 14 to 98 points, with higher scores indicating higher levels of knowledge regarding EBP. At the time of its development, the Cronbach’s alpha of the tool was 0.91 [35] and 0.93 for Korean nurses [19]. In this study, the Cronbach’s alpha was found to be 0.83.

#### EBP beliefs

Participants’ beliefs in valuing EBP were measured using the Evidence-based Practice Beliefs (EBPB) tool, developed by Melnyk and colleagues [36]. This tool consists of 16 questions. Examples of the items in the EBPB include “I am sure that I can implement EBP in a time efficient way” and “I am sure about how to measure the outcomes of clinical care.” Each question is rated on a five-point Likert scale (1 = strongly disagree, 5 = strongly agree), but scoring for items 11 and 13 was reversed. Possible total scores range from 16 to 80 points, with higher scores indicating positive EBP beliefs. At the time of its development, the Cronbach’s alpha of the tool was 0.90 [36] and 0.88 for Korean nurses [34]. In this study, the Cronbach’s alpha was found to be 0.81.

#### Organizational readiness for EBP

The organization’s culture and its readiness for system-wide EBP implementation were measured using the Organizational Culture and Readiness Scale for System-Wide Integration of Evidence-Based Practice (OCRSIEP) [27]. The OCRSIEP scale was developed to measure the levels of readiness in performing EBP, at the organizational level, and consists of 25 questions that offer insights into the strengths of and opportunities related to fostering EBP. Possible total scores range from 25 to 125 points, indicating that the higher the score, the better the organizational readiness and cultural cultivation for implementing EBP. The following questions are asked: “To what extent is EBP clearly described as central to the mission and philosophy of your institution?” and “To what extent is the nursing staff with whom you work committed to EBP?” At the time of its development, the Cronbach’s alpha of the tool was 0.94 [27] and 0.95 for Korean nurses [22]. In this study, the Cronbach’s alpha was found to be 0.87.

#### EBP implementation

The frequency of performing EBP-related activities was measured using the Evidence-Based Practice Implementation tool, developed by Melnyk and colleagues [36]. This tool consists of 18 questions pertaining to how often, in the last 8 weeks, participants performed certain EBP activities, such as “Generated a PICOT (P = patient, I = intervention, C = comparison, O = outcome, T = time) question about my clinical practice,” “Accessed the National Guidelines Clearinghouse,” and “Evaluated a care initiative by collecting patient outcome data.” Responses on the scale range from 0 (0 times) to 4 (over 8 times). The possible total scores range from 0 to 72 points, with higher scores indicating higher levels of commitment to implementing EBP-related activities. At the time of its development, the Cronbach’s alpha of the tool was 0.96 [36] and 0.95 for Korean nurses [22]. In this study, the Cronbach’s alpha was found to be 0.81.

### Data collection and ethical considerations

The Institutional Review Board (IRB) approval was obtained prior to data collection from the authors’ institution (no. 2-1041055-AB-N-01-2018-10, Chosun University Institutional Review Board). Data were collected from December 2017 to January 2018. For data collection, we contacted a nurse administrator at Chosun University Hospital and explained the purpose of this study. Chosun University Hospital is a private university-affiliated, tertiary care hospital located in Gwangju city, South Korea. It is located in Gwangju Metropolitan City in the southern district of Korea and is in charge of medical services in Jeolla province. The hospital consists of 849 beds, with 25 medical departments in operation, including 15 general wards, 4 intensive care units, regional emergency medical center, operating rooms, outpatient departments, and laboratories. A researcher visited the hospital to explain the purpose of this study as well as the inclusion criteria to the nurse unit managers, during a supervisor meeting. The questionnaires were enclosed in different envelopes for each ward and distributed by the staff and assistants of the nursing education team who did not participate in this survey. The collection boxes were made and distributed to each ward, and nurses were allowed to submit questionnaires voluntarily at any time. To ensure anonymity of the participants, the consent form was given in writing with a mark or numbers that could only be known to themselves. Therefore, all nurses, whose questionnaires were collected, were considered to have participated in this study of their own will.

### Statistical analysis

Data analysis was performed using SPSS V 25.0. Descriptive statistics, including the means, standard deviations, frequencies, and percentages, were used to describe the participants’ general and research-related characteristics, and EBP-related variables. Differences between major variables, by participants’ characteristics, were analyzed through independent t-tests, analysis of variance, and Scheffe test. The relationships between major variables were analyzed using Pearson’s correlation coefficient. Hierarchical multiple regression was performed to determine the factors affecting the implementation of EBP.

## Results

### General and research-related characteristics of the participants

Table 1 presents the participants’ characteristics. The mean age of all participants was 31.9±9.2 years, with 58.9% aged 21 to 30. Their overall clinical experience was 9.0+4.2 years, with 80.1% working as staff nurse. Approximately 80.5% of the participants had an associate or bachelor’s degree in nursing, 80.1% worked as staff nurses, and 59.1% worked in a general ward. Approximately 73.5% and 59.7% of nurses completed nursing research and statistics classes, respectively, but most of them answered that they only completed their undergraduate courses. While 46.6% of nurses had experience taking EBP classes, only 25.7% were familiar with EBP-related terms.

**Table 1:** General and research-related characteristics of the participants.

Variables	n (%)	Variables	n (%)
**Gender**		**Working unit**	
Women	486(93.3)	General ward	308(59.1)
Men	35(6.7)	Intensive care unit	80(15.4)
**Age** (Years; Mean, SD)	(31.9, 9.2)	Emergency room	40(7.7)
≤25	158(30.3)	Operation room/recovery room	43(8.2)
26–30	149(28.6)	Outpatient	48(9.2)
31–35	79(15.2)	Nursing administration	2(0.4)
36–40	36(6.9)	**Completed nursing research course**	
≥41	99(19.0)	Yes	383(73.5)
**Clinical experience** (Mean, SD)	(9.03, 4.2)	No	138(26.5)
≤12 months	96(18.4)	**Completed statistics course**	
13-36months	105(20.3)	Yes	311(59.7)
37-60months	40(7.7)	No	210(40.3)
61-120months	117(22.5)	**Attendance of EBP lecture**	
121-240months	74(14.2)	Yes	243(46.6)
≥241months	88(16.9)	No	278(53.4)
**Educational level**		**Research conducting/participated**	
Diploma/associates	77(14.8)	Yes	147(28.2)
Bachelors	343(65.7)	No	374(71.8)
Masters or ongoing	93(17.9)	**Membership of academic society**	
Doctors or ongoing	8(1.6)	Yes	41(7.9)
**Position**		No	480(92.1)
Staff nurse	418(80.1)	**Attendance of academic conference**	
Charge nurse	42(8.1)	Yes	72(13.8)
Advanced nurse practitioner	28(5.4)	No	449(86.2)
Head nurse	29(5.6)	**Familiar to EBP terminology**	
Team manager/administrator	4(0.8)	Yes	134(25.7)
		No	387(74.3)

Abbreviations: EBP = evidence-based practice

### Level of EBP knowledge, beliefs, organizational readiness, and EBP implementation

The level of EBP knowledge was 52.5 ± 11.1 points out of 98. Participants were highly knowledgeable on the use of information technology to search for and use data (4.3 ± 0.9) and shared these ideas and information with colleagues (4.3 ± 1.0). However, they have less knowledge on how to convert these data into research problems (3.8 ± 1.0) and critically analyze existing evidence (3.8 ± 0.9).

The level of EBP beliefs, among the participants, was relatively positive, with a total score of 51.7 ± 5.9 points out of 80. The items with the highest score were “I am sure that evidence-based guidelines can improve clinical care” (3.8 ± 0.6) and “I am sure that implementing EBP will improve the care that I deliver to my patients” (3.7 ± 0.6). The items with the lowest scores were “I believe EBP is difficult” (2.5 ± 0.6) and “I believe that EBP takes too much time” (2.7 ± 0.6).

The level of the organizational readiness to perform EBP perceived by nurses was 76.4 ± 13.0 points out of 125. The participants demonstrated lowest level of readiness on the following aspects: “To what extent are decisions generated from direct care providers” (2.7 ± 0.8) and “To what extent are librarians used to search for evidence” (2.7 ± 0.7). The participants demonstrated high levels of readiness on the following aspect: “To what extent are decisions generated from upper administration” (3.7 ± 0.8) and “To what extent are there EBP champions in the environment among administrators” (3.3 ± 0.7).

The level of EBP implementation was 15.0 ± 3.2 points out of 72. The items with the lowest scores were “accessed the National Guideline Clearinghouse” (0.4 ± 0.2) and “accessed the Cochrane database of systematic reviews” (0.4 ± 0.2). Participants had low levels of engagement in the following activities: “used an EBP guideline or systematic review to change clinical practice where I work” (0.6 ± 0.2) and “shared evidence from studies to over 2 colleagues” (0.7 ± 0.2). Items were listed in order, and details are given in Table 2 and S1 Appendix.

**Table 2:** Level of EBP knowledge, beliefs, organizational readiness and EBP implementation.

Items	Mean, SD
**EBP knowledge**	52.5, 11.1
**EBP beliefs**	51.7, 5.9
**Organizational readiness for EBP**	76.4, 13.0
**EBP implementation**	15.0, 3.2

Abbreviations: EBP = evidence-based practice

### Differences in the levels of EBP knowledge, beliefs, organizational readiness, and EBP implementation according to participant characteristics

Table 3 presents the differences in EBP variables according to the participants’ characteristics. The level of EBP knowledge significantly differed by age (F = 5.542), clinical experience (F = 4.545), position (F = 9.292), educational level (F = 5.084), and research-related activities. The level of EBP beliefs significantly differed by age (F = 5.370), clinical experience (F = 2.653), position (F = 9.142), educational level (F = 4.585), and research-related activities. Organizational readiness significantly differed by age (F = 13.149), clinical experience (F = 12.814), educational level (F = 5.132), attendance to EBP lectures (t = 2.191), research conducted or research participation (t = 4.033), and familiarity to EBP terminologies (t = 4.062). In terms of characteristics by units, nursing administrators with decision-making authority recognized that the organization’s readiness (F = 3.626) was relatively low compared with that of staff nurses. The level of EBP implementation was mainly related to the experience of statistics courses (t = 2.004), attendance to EBP lectures (t = 2.069), research conducted or research participation (t = 2.953), and familiarity to EBP terminologies (t = 2.508). In terms of characteristics by units, nursing administrators recognized that the level of EBP implementation (F = 2.385) were relatively low compared to staff nurses.

**Table 3:** Differences in the levels of EBP beliefs, knowledge, organizational readiness, and EBP implementation according to the participants characteristics.

Variables	Category	EBP* Knowledge *M*, *SD*	*t / F (p)*	EBP Beliefs *M*, *SD*	*t / F* (*p*)	Organizational Readiness *M*, *SD*	t / *F* (*p*)	EBP Implementation *M*, *SD*	*t / F* (*p*)
**Age (years)**	≤25 ^a^	55.1, 9.8	5.524	51.7, 5.3	5.370	82.2, 11.5	13.149	15.3, 11.1	.365
	26–30 ^b^	57.4, 10.1	(< .001)	50.4, 5.8	(< .001)	75.4, 12.1	(< .001)	15.1, 10.2	(.834)
	31–35 ^c^	56.5, 11.1	^e>a^	51.1, 6.6	^e>b^	72.1, 13.4	^a>b,c,d,e^	16.0, 11.4	
	36–40 ^d^	60.4, 13.6		52.6, 6.1		74.1, 13.0		14.1, 10.3	
	≥41 ^e^	61.2, 12.3		53.8, 5.9		72.8, 13.5		14.1, 10.2	
**Clinical experience (months)**	≤12 ^a^	53.8, 10.1	4.545	51.2, 5.3	2.653	82.6, 11.4	12.814	14.5, 11.7	.984
	13–36 ^b^	57.1, 9.7	(< .001)	51.9, 5.8	(.022)	80.7, 11.6	(< .001)	15.4, 12.0	(.427)
	37–60 ^c^	56.1, 9.1	^f>a^	50.4, 6.5		76.8, 11.9	^a,b >d,e,f^	13.1, 10.2	
	60–120 ^d^	57.9, 10.8		50.9, 5.7		72.6, 12.9		16.9, 13.2	
	121–240 ^e^	58.3, 12.3		51.9, 7.1		71.6, 12.2		14.7, 11.6	
	≥241 ^f^	61.1, 12.4		53.5, 5.5		73.3, 13.6		13.9, 12.4	
**Position**	Staff nurse ^a^	56.3, 10.4	9.292	51.1, 5.7	9.142	77.0, 12.7	2.123	15.0, 11.8	.432
	Charge nurse ^b^	61.1, 13.0	(< .001)	53.0, 6.6	(< .001)	73.7, 13.8	(.096)	14.3, 12.3	(.730)
	Nurse practitioner ^c^	65.2, 10.7	^c>a^	55.0, 7.4	^c,d>a^	77.0, 15.1		17.5, 16.2	
	Head nurse/Team leader ^d^	61.3, 12.2		55.2, 4.4		72.0, 13.5		14.8, 11.8	
**Working unit**	General ward ^a^	57.2, 10.5	.993	51.9, 6.1	1.442	77.3, 13.1	3.626	14.2, 12.0	2.385
	Intensive care unit ^b^	57.5, 11.5	(.421)	50.7, 6.2	(.208)	76.4, 12.6	(.003)	18.2, 11.2	(.037)
	Emergency room ^c^	55.8, 12.9		50.9, 5.8		75.2, 12.0	^a >f^	16.7, 14.5	
	Outpatient ^d^	57.7,11.1		53.1, 5.0		76.6, 13.3		12.6, 10.8	
	Operating/Recovery room ^e^	60.3, 12.0		51.0, 5.6		71.8, 12.3		17.0, 12.3	
	Nursing administration ^f^	65.5, 10.6		55.5, 7.7		46.0, 9.8		5.5, 3.5	
**Education**	Diploma/associate ^a^	57.6, 10.5	5.084	51.5, 5.9	4.585	76.7, 12.6	5.123	17.1, 11.6	1.065
	Bachelors ^b^	56.5, 10.7	(.002)	51.2, 5.8	(< .001)	77.6, 12.8	(.002)	14.9, 12.4	(.363)
	Masters or ongoing ^c^	60.0, 12.0	^d> b^	53.3, 6.3	^c>b^	72.7, 12.9	^c>b^	13.8,11.5	
	Doctors or ongoing ^d^	68.1, 9.9		56.3, 3.9		66.3, 17.9		14.2, 11.3	
**Completed nursing research course**	Yes	57.5, 11.0	.484	51.7, 5.9	.549	76.8, 12.8	1.476	15.3, 12.2	1.159
	No	57.0, 10.8	(.643)	51.4, 6.0	(.583)	74.9, 13.4	(.141)	13.9, 11.8	(.247)
**Completed statistics course**	Yes	58.7, 11.0	2.994	52.1, 5.9	1.744	76.6, 12.6	.536	15.9, 12.2	2.004
	No	55.7, 11.0	(.003)	51.1, 6.0	(.082)	76.0, 13.7	(.529)	13.7, 11.9	(.046)
**Attendance of EBP lecture**	Yes	58.1, 11.9	1.271	52.4, 6.1	2.528	77.7, 13.2	2.191	16.2, 12.8	2.069
	No	56.9, 10.3	(.204)	51.1, 5.8	(.012)	75.1, 12.7	(.029)	14.0, 11.4	(.039)
**Conducting research or**	Yes	59.6, 11.4	2.824	52.6, 5.8	2.276	80.0, 12.6	4.033	17.5, 13.2	2.953
**Research participation**	No	56.6, 10.8	(.005)	51.3, 6.0	(.023)	74.9, 12.9	(< .001)	14.0, 11.5	(.003)
**Membership of academic**	Yes	63.2, 12.1	3.485	54.7, 5.5	3.403	76.4, 12.0	.021	15.3, 12.9	.123
**society**	No	57.0, 10.8	(.001)	51.4, 5.9	(.001)	76.4, 13.1	(.983)	15.0, 12.1	(.902)
**Attendance of academic**	Yes	62.5, 13.4	3.497	53.8, 6.5	3.179	76.1, 12.3	.228	15.8, 13.7	.567
**conference regularly**	No	56.7, 10.4	(.001)	51.4, 5.8	(.002)	76.4, 13.0	(.820)	14.9, 11.9	(.571)
**Familiar to EBP terminology**	Yes	61.0, 11.6	4.299	54.7, 5.4	7.127	80.3, 13.1	4.062	17.3, 13.2	2.508
	No	56.3, 10.6	(< .001)	50.6, 5.0	(< .001)	75.0, 12.8	(< .001)	14.3, 11.6	(.012)

### Relationship among EBP knowledge, beliefs, organizational readiness, and EBP implementation

Bivariate Pearson’s correlation analysis showed that EBP implementation had a significantly positive correlation with EBP knowledge (r = .304, p < .001), beliefs (r = .272, p < .001), and organizational readiness (r = .430, p < .001). In addition, EBP knowledge were statistically positively correlated with EBP beliefs (r = .555, p < .001) and organizational readiness (r = .314, p < .001). EBP beliefs and organizational readiness were statistically positively correlated (r = .406, p < .001) (Table 4).

**Table 4:** Correlation between EBP knowledge, beliefs, organizational readiness and EBP implementation.

Variables	Pearson’s correlation coefficients, r*		
	EBP knowledge	EBP beliefs	Organizational readiness
**EBP knowledge**	1.000	-	-
**EBP beliefs**	.555	1.000	-
**Organizational readiness**	.314	.406	1.000
**EBP implementation**	.304	.272	.430

Abbreviations: EBP = evidence-based practice.

*p < .001 for all the Pearson’s correlation coefficients in the table.

### Factors affecting EBP implementation

In the final model, EBP knowledge (β = .15) and organizational readiness (β = .36) were significant predictors of EBP implementation; the model predicted 22.2% of the variance in EBP implementation (F = 10.098, p < .001) (Table 5). Age was highly correlated with clinical experience and was excluded from independent variables. Prior to the regression analysis, the data were checked for multicollinearity using tolerance (0.366–0.911) and the variance inflation factor (1.183–2.733). Variance inflation factor values greater than 10 and tolerance-values smaller than 0.10 may indicate multicollinearity. The Durbin-Watson value was 1.905, and each model demonstrated good statistical values.

**Table 5:** Factors affecting EBP implementation.

Variables	Model 1*			Model 2*			Model 3*			Model 4*			
	b	β	p	b	β	p	b	β	p	b	β	p	VIF
Clinical experience	-.01	-.06	.342	-0.1	-.07	.246	-.00	-.06	.322	.00	.03	.558	2.733
Educational level	-2.35	-.21	.028	-2.16	-.11	.036	-2.26	-.11	.027	-1.76	-.09	.069	1.576
Position	1.38	.09	.132	.94	.06	.285	.68	.04	.442	.49	.03	.558	2.231
Working unit	.36	.04	.402	.34	.03	.400	.42	.04	.303	.65	.07	.097	1.195
Completed nursing research course	.69	.02	.671	.01	.00	.994	-.07	-.00	.961	.60	.02	.684	1.838
Completed statistics course	-2.30	-.09	.113	-1.37	-.05	.329	-1.37	-.05	.325	-1.96	-.08	.138	1.826
Attendance EBP lecture	-.02	-.00	.982	-.25	-.01	.835	-.20	-.00	.870	.55	-.02	.630	1.446
Conducting research	-3.09	1.11	.018	-2.39	-.08	.059	-2.39	-.08	.057	-1.09	-.04	.364	1.273
Membership of academic society	1.11	.02	.635	1.76	.03	.46	2.04	.04	.363	2.21	.04	.298	1.385
Attendance of academic conference regularly	.12	.00	.946	.68	.01	.701	.59	.01	.740	.68	.01	.686	1.445
Familiar to EBP terminology	-2.16	-.07	.109	-1.18	-.04	.358	-.46	-.01	.723	.28	.01	.819	1.272
EBP knowledge				.31	.28	.000	.23	.21	.000	.16	.15	.002	1.526
EBP belief							.29	.14	.006	.05	.02	.597	1.707
Organizational readiness										.33	.36	.000	1.430
R^2^ (⊿R^2^)	.045			.118 (.073)			.131 (.013)			.222 (.091)			
F (p)	2.111 (.018)			5.521 (< .001)			5.738 (< .001)			10.098 (< .001)			

Abbreviations: EBP = evidence-based practice

* Model 1: General, research-related Characteristics, Model 2: General, research-related Characteristics, EBP knowledge, Model 3: General, research-related Characteristics, EBP knowledge, EBP beliefs, Model 4: General, research-related Characteristics, EBP knowledge, EBP beliefs, Organizational readiness.

## Discussion

EBP knowledge, beliefs, and organizational readiness were significantly correlated with EBP implementation and hierarchical regression presented them as major predictors. Model 1 of regression shows that completing a postgraduate or higher curricula and conducting or participating in research had a significant impact on the level of EBP implementation. In Models 2 and 3 of EBP knowledge, beliefs, and organizational readiness, each variable had a significant effect. In Model 4, EBP knowledge and organizational readiness were the main influencing factors on EBP implementation. Based on these findings, the successful implementation of EBP should prioritize efforts to establish an education strategy to improve EBP knowledge and to create an organizational culture for preparing and supporting EBP at the nursing organization level.

In this study, the EBP implementation level was 15.0 out of 72 points. Melnyk et al.’s study [37] reported an implementation level of 18.9 points, while that in Korea study [26] was 33.0 points, which were relatively higher than that reported in this study. A previous study of 410 nurses working at 10 tertiary hospitals in Korea also showed an average implementation level of 0.95 points [22]. Considering that the EBP implementing scores in this study ranged from zero (when there has been no EBP-related activity over the past 8 weeks) to 1 point (when EBP-related activities were performed one or three times) [36], suggests that EBP implementation has not been activated at the actual clinical setting. This shows that in South Korea, EBP are only implemented around major large-scale tertiary hospitals located in the Seoul metropolitan area, and the spread and implementation of EBP to a wide range of areas and smaller hospitals, including provincial cities, is insufficient [17–19, 22]. Previous studies in South Korea also pointed out the spread of EBP concentrated only in Seoul and its surrounding metropolitan areas and stressed the need for efforts to overcome these limitations [17,18].

In recent studies [18, 22, 23], the lack of knowledge among nurses regarding research and statistical methods, education, and lack of experience in research and statistics are reported as important predictors of a nurse’s poor performance of EBP. These studies also point out the overall lack of clinical inquiry creation, accessing and searching for evidence, and critical appraisal and practical application by nurses. EBP experts emphasize that nurses should be sensitive to the continuously generated scientific knowledge and have sufficient knowledge to make critical judgments about such research in order to perform EBP [12, 15, 24, 25]. However, EBP knowledge is difficult to improve by nurses' personal efforts alone, so organizational support is needed [34]. Several previous studies suggest the necessity of learning atmospheres and facilities for successful EBP establishment in nursing organizations [12, 21, 38, 39]. Beyond acquiring knowledge at the individual level, in order to efficiently acquire the knowledge necessary for decision making at the clinical setting, organizations need a system of knowledge management, information systems and databases related to nursing. Encouraging organizational team learning and continuing learning opportunities to improve nurses’ EBP knowledge can be used as an educational strategy. Cho et al. [38] emphasized using a learning organization as an alternative rather than traditional lecturing methods as a way of delivering knowledge. To do this, it is necessary to operate a ward-level education program in the form of workshops, which includes discussions and brainstorming sessions, rather than a lecture-style education. It is also necessary to establish customized education strategies, such as the operation of journal clubs in wards considering clinical topics specified by each ward. A learning organization that includes an EBP preceptor or EBP mentoring program may also be considered. Introducing and providing the EBP concept in the preceptor training program at the organizational level, helps to have knowledge and positive beliefs about EBP. In addition, it will be necessary to motivate nurses to participate in the conference and to provide incentives for them to present at the conference. Human resources such as EBP mentors will need to be trained so that they can serve as EBP facilitators and EBP champions in wards. In addition, when developing EBP education programs, a hands-on education must be developed and implemented in collaboration with librarians so that practical performance can now be carried out beyond the EBP concept or its importance should be emphasized. Nam et al. [39] reported that EBP education programs, which include intensive training such as a four-hour workshop per day, computer-based training consisting of a total of three modules that take 15–20 minutes per module, and team-based training programs of 2–3 hours per week, were effective in improving EBP knowledge. One option would be to develop EBP-related cases for each clinical scenario, and to operate a simulation-based EBP training program.

Along with educational strategies for improving EBP knowledge, the establishment of organizational cultural development and support strategies should be considered [10, 24]. Organizational readiness for EBP in this study (76.4 points out of 125) was relatively low compared to a recent large online survey conducted in the US (80.2 points) [37]. In Korea, direct comparisons are limited due to the use of different tools. In the study of Cho et al. [17], the level of organization support was 3.7 points out of a 5-point scale and 3.3 points as reported by Kim et al. [40]. In particular, the items with the lowest level of organizational readiness in this study reported lack of the decision-making authority of clinical nurses who perform direct care, lack of support personnel such as librarians, and lack of budget support of nursing organizations to perform EBP. These results suggest that it is urgent to create a nursing organizational culture that facilitates access and utilization of EBP within clinical settings, and prepare all clinical nurses for the successful implementation of the EBP [8, 41]. The ARCC^©^ model emphasizes that in order to establish the concept of EBP in organizational culture, the contents of EBP must be clearly stated in the organization’s mission and vision, and consensus on common values. It also emphasizes the need for human resources such as EBP mentors to facilitate EBP, along with improvements in the physical environment [24]. In this study, there is a shortage of human resources, such as nursing researchers with doctoral degrees or higher, or educators with expertise in EBP, and nurses providing direct care have limited participation and authority in the decision-making in the organization, which requires active intervention and support at the organization level. It is necessary to form an independent EBP committee within the nursing organization for the facilitation of EBP and to establish and implement policies for the creation of EBP culture by leading this committee.

To create such an organizational culture, it is necessary to understand the characteristics of Korean nursing organizations. In general, south Korea has a higher nurse-to-patient ratio, a relatively high working time for nurses in three shifts, and a high workload compared with other countries such as the US, UK and Canada [42, 43]. Additionally, South Korea’s nursing organizational culture has a tendency to be mainly hierarchical compared with other Western countries, and this vertical structure has been shown to reduce nurses’ work performance, professional autonomy, job satisfaction, and willingness to serve compared with other organizational cultures [16, 22, 42–44]. The ARCC^©^ model emphasizes the creation of an EBP culture that facilitates clinical inquiry as part of the EBP facilitation strategy [24]. This culture emphasizes the flexibility of the nursing organization to respond to the rapidly changing environment and supports nurses questioning existing nursing practices with professional autonomy [7, 24]. In this culture, nurses can perform a series of EBP steps to create various clinical questions, search for evidence, and critically evaluate and apply to practice [45]. Therefore, the willingness and leadership of nursing administrators with decision-making authority to facilitate EBP implementation are very important.

After analyzing the differences in major variables according to the characteristics of the nurses, higher educational status and experiences of conducting or participating in research had a significant impact on EBP implementation. The more research-related activities, the higher the level of EBP knowledge, beliefs, and organizational readiness, and the higher the level of EBP implementation [17, 22, 26, 34, 46]. It is necessary to provide both nursing managers and staff nurses with the opportunity to participate directly in the process of planning and carrying out EBP-related research projects at actual clinical settings [21]. Through this, it is necessary to reduce the unfamiliarity with EBP and to support frequent positive experiences through direct activities.

The results of this study will contribute to establishing systematic education/training programs and provide the basis for fostering EBP cultures for the successful implementation of the EBP, but there are some limitations. The level of EBP implementation can be affected by various factors, including the type of organizational culture, the characteristics of each hospital organization, regional characteristics, the type of leadership by units, and the composition of nursing staff. Therefore, in future studies, it is expected that the variance of the regression model will be improved by considering these variables. Moreover, as this survey was conducted at one particular hospital located in Korea, the results of this study cannot be generalized. Despite these limitations, we expect that active implementation of these strategies will contribute to providing a stepping stone for the next phase of EBP.

## Conclusions

The results of this study suggest that the level of organizational readiness is the greatest factor in EBP implementation. Based on these results, the main focus of the study was the importance of individual nurses’ efforts in carrying out EBP, but above all efforts to create an organizational culture to prepare and support EBP at the nursing organization level. While the performance of EBP positively improves nursing-sensitive outcomes, the process of establishing such EBP also creates a work and psychological burden for clinical nurses and can also lead to resistance from unfamiliar concepts [10]. The hospital where this study was conducted has not yet activated EBP, but there has been a high demand for nursing managers and nurses to accept the new concept of EBP.

In the initial process of introducing and establishing EBP, nursing organizations will need to minimize expected barriers, enhance facilitators, and strive to build an infrastructure that includes vision, policy-making, budgeting, excellent personnel and facilities within the organization. In addition, it is necessary to participate in ongoing education training, as the improvement of individual EBP knowledge among nurses can enhance positive beliefs and values regarding EBP and actual performance. To this end, of course, the nursing administration will need to develop a curriculum that will foster and evaluate the EBP knowledge of each nurse.

## Supporting information

S1 Appendix Table 2. Level of EBP knowledge, beliefs, organizational readiness and EBP implementation.
(DOCX)

S2 Appendix The original questionnaire (Korean version). (PDF)

S1 Dataset Data of questionnaire. (XLSX)
