# Supplementary material for: Clinical nurses’ beliefs, knowledge, organizational readiness and level of implementation of evidence-based practice: The first step to creating an evidence-based practice culture
Source: PLoS One. 2019 Dec 26;14(12):e0226742. doi: 10.1371/journal.pone.0226742 (PMC6932768; doi:10.1371/journal.pone.0226742)
Supplement: S1 Appendix — Level of EBP knowledge, beliefs, organizational readiness and EBP implementation. (DOCX) [file pone.0226742.s001.docx]

| **Appendix 1**  **Table 2. Level of EBP knowledge, beliefs, organizational readiness and EBP implementation.** | | | |
| --- | --- | --- | --- |
| **Items** | | **Mean, SD** | **Rank** |
| **EBP knowledge** |  | 52.5, 11.1 |  |
| IT skills. | | 4.3, 0.9 | 1 |
| Sharing ideas and information with colleagues. | | 4.3, 1.0 | 2 |
| Awareness of major information types and sources. | | 4.1, 0.9 | 3 |
| Monitoring and reviewing of practice skills. | | 4.1, 0.9 | 4 |
| Dissemination of new ideas about care to colleagues. | | 4.1, 1.0 | 5 |
| Ability to review your own practice. | | 4.1, 0.9 | 6 |
| Ability to determine how useful (clinically applicable) the material is. | | 4.1, 0.9 | 7 |
| Ability to apply information to individual cases. | | 4.1, 1.0 | 8 |
| Knowledge of how to retrieve evidence. | | 4.1, 1.0 | 9 |
| Ability to identify gaps in your professional practice. | | 4.0, 0.9 | 10 |
| Ability to determine how valid (close to the truth) the material is. | | 4.0, 0.9 | 11 |
| Research skills. | | 3.9, 1.0 | 12 |
| Ability to analyze critically evidence against set standards. | | 3.8, 0.9 | 13 |
| Converting your information needs into a research question. | | 3.8, 1.0 | 14 |
| **EBP beliefs** | | 51.7, 5.9 |  |
| I am sure that evidence-based guidelines can improve clinical care. | | 3.8, 0.6 | 1 |
| I am sure that implementing EBP will improve the care that I deliver to my patients. | | 3.7, 0.6 | 2 |
| I believe that EBP results in the best clinical care for patients. | | 3.6, 0.6 | 3 |
| I believe that critically appraising evidence is an important step in the EBP process. | | 3.4, 0.7 | 4 |
| I believe the care that I deliver is evidence-based. | | 3.3, 0.6 | 5 |
| I am sure that I can implement EBP in a time efﬁcient way. | | 3.3, 0.6 | 6 |
| I believe that I can search for the best evidence to answer clinical questions in a time  efﬁcient way. | | 3.2, 0.6 | 7 |
| I believe that I can overcome barriers in implementing EBP. | | 3.2, 0.6 | 8 |
| I am sure that I can access the best resources in order to implement EBP. | | 3.2, 0.6 | 9 |
| I am conﬁdent about my ability to implement EBP where I work. | | 3.2, 0.6 | 10 |
| I am sure that I can implement EBP. | | 3.1, 0.6 | 11 |
| I am sure about how to measure the outcomes of clinical care. | | 3.0, 0.7 | 12 |
| I know how to implement EBP sufﬁciently enough to make practice changes. | | 2.9, 0.7 | 13 |
| I am clear about the steps of EBP. | | 2.9, 0.7 | 14 |
| I believe that EBP takes too much time. | | 2.7, 0.6 | 15 |
| I believe EBP is difficult. | | 2.5, 0.6 | 16 |
| **Organizational readiness for EBP** | | 76.4,13.0 |  |
| To what extent are decisions generated from upper administration. | | 3.7, 0.8 | 1 |
| To what extent are there EBP champions in the environment among administrators. | | 3.3, 0.7 | 2 |
| To what extent do staff nurses have proficient computer skills. | | 3.2, 0.7 | 3 |
| To what extent is EBP clearly described as central to the mission and philosophy of your institution. | | 3.2, 0.7 | 4 |
| To what extent are decisions generated from physician or other healthcare provider  groups. | | 3.2, 0.8 | 5 |
| To what extent are there EBP champions in the environment among nurse educators. | | 3.1, 0.7 | 6 |
| To what extent are there EBP champions in the environment among physicians. | | 3.1, 0.7 | 7 |
| To what extent is the nursing staff with whom you work committed to EBP. | | 3.1, 0.7 | 8 |
| To what extent are there EBP champions in the environment among advanced nurse  practitioners. | | 3.1, 0.7 | 9 |
| In your organization, to what extent is there a critical mass of nurses who have strong  EBP knowledge and skills. | | 3.1, 0.8 | 10 |
| To what extent do you believe that EBP is practiced in your organization. | | 3.1, 0.6 | 11 |
| To what extent is the physician team with whom you work committed to EBP. | | 3.1, 0.7 | 12 |
| To what extent are there EBP champions in the environment among staff nurses. | | 3.0, 0.7 | 13 |
| To what extent is the measurement and sharing of outcomes part of the culture of the organization in which you work. | | 3.0, 0.7 | 14 |
| In your organization, to what extent are there advanced nurse practitioners who are EBP mentors for staff nurses as well as other advanced nurse practitioners. | | 3.0, 0.8 | 15 |
| To what extent do practitioners model EBP in their clinical settings. | | 2.9, 0.7 | 16 |
| To what extent are administrators within your organization committed to EBP. | | 2.9, 0.7 | 17 |
| To what extent are there nurse scientists (doctorally prepared researchers) in your organization to assist in generation of evidence when it does not exist. | | 2.9, 0.7 | 18 |
| To what extent are librarians within your organization have EBP knowledge and skills. | | 2.9, 0.7 | 19 |
| To what extent do staff nurses have access to quality computers and access to electronic databases for searching for best evidence. | | 2.8, 0.7 | 20 |
| Compared to 6months ago, how much movement in your organization has there been  toward EBP culture. | | 2.8, 0.8 | 21 |
| Overall, how would you rate your institution in readiness for EBP. | | 2.8, 1.0 | 22 |
| To what extent are fiscal resources used to support EBP. | | 2.8, 0.8 | 23 |
| To what extent are librarians used to search for evidence. | | 2.7, 0.7 | 24 |
| To what extent are decisions generated from direct care providers. | | 2.7, 0.8 | 25 |
| **EBP implementation** |  | 15.0, 3.2 |  |
| Used evidence to change my clinical practice. | | 1.2, 0.2 | 1 |
| Critically appraised evidence from a research study. | | 1.0, 0.2 | 2 |
| Shared an EBP guideline with a colleague. | | 0.9, 0.2 | 3 |
| Collected data on a patient problem. | | 0.9, 0.2 | 4 |
| Informally discussed evidence from a research study with a colleague. | | 0.9, 0.3 | 5 |
| Shared the outcome data collected with colleagues. | | 0.9, 0.3 | 6 |
| Generated a PICO question about my clinical practice. | | 0.9, 0.2 | 7 |
| Evaluated the outcomes of a practice change. | | 0.9, 0.2 | 8 |
| Changed practice based on patient outcome data. | | 0.8, 0.2 | 9 |
| Shared evidence from a research study with a multidisciplinary team member. | | 0.8, 0.2 | 10 |
| Evaluated a care initiative by collecting patient outcome data. | | 0.8, 0.2 | 11 |
| Shared evidence from are search study with a patient/family member. | | 0.8, 0.2 | 12 |
| Promoted the use of EBP to my colleagues. | | 0.7, 0.2 | 13 |
| Read and critically appraised a clinical research study. | | 0.7, 0.2 | 14 |
| Shared evidence from studies to over 2 colleagues. | | 0.7, 0.2 | 15 |
| Used an EBP guideline or systematic review to change clinical practice where I work. | | 0.6, 0.2 | 16 |
| Accessed the Cochrane database of systematic reviews. | | 0.4, 0.2 | 17 |
| Accessed the National Guideline Clearinghouse. | | 0.4, 0.2 | 18 |
| Abbreviations: EBP = evidence-based practice; PICO = P(patient), I(intervention), C(comparison), O(outcome). | | | |
