# Supplementary material for: Clinical nurses’ beliefs, knowledge, organizational readiness and level of implementation of evidence-based practice: The first step to creating an evidence-based practice culture
Source: PLoS One. 2019 Dec 26;14(12):e0226742. doi: 10.1371/journal.pone.0226742 (PMC6932768; doi:10.1371/journal.pone.0226742)
Supplement: S2 Appendix — (PDF) [file pone.0226742.s002.pdf]

## S1 Fig. The original questionnaire (Korean version).

※ 다음은 근거기반실무에 대한 신념을 묻는 질문입니다. 정답이나 오답은 없습니다. 각 문항을 읽고 귀하의 생각과 가장 가까운 곳에 V 표시 해주세요.

| 문 항 | 매우 부정 | 부정 | 보통 | 긍정 | 매우 긍정 |
|---|---|---|---|---|---|
| 1. 나는 **근거기반실무가 환자들에게 최상의 간호를 제공**한다고 믿는다. | 1 | 2 | 3 | 4 | 5 |
| 2. 나는 **근거기반실무의 단계**를 명확하게 알고 있다. | 1 | 2 | 3 | 4 | 5 |
| 3. 나는 **내가 근거기반실무를 실행할 수 있다**고 확신한다. | 1 | 2 | 3 | 4 | 5 |
| 4. 나는 **근거를 비판적으로 평가하는 것**이 근거기반실무의 중요한 단계라고 믿는다. | 1 | 2 | 3 | 4 | 5 |
| 5. 나는 **근거기반 실무지침이 임상 간호를 향상시킨다**고 생각한다. | 1 | 2 | 3 | 4 | 5 |
| 6. 나는 내가 임상질문에 적합한 **최상의 근거를 빠른 시간 내에 효율적으로 검색할 수 있다**고 믿는다. | 1 | 2 | 3 | 4 | 5 |
| 7. 나는 근거기반실무를 수행하는데 **장애가 되는 요소들을 내가 극복할 수 있다**고 믿는다. | 1 | 2 | 3 | 4 | 5 |
| 8. 나는 근거기반실무를 빠른 시간 내에 **효율적으로 수행할 수 있다**고 확신한다. | 1 | 2 | 3 | 4 | 5 |
| 9. 나는 근거기반실무를 적용하는 것이 내가 **환자들에게 제공하는 간호를 향상시킬 것**이라고 믿는다. | 1 | 2 | 3 | 4 | 5 |
| 10. 나는 **임상간호의 결과를 어떻게 측정하는지** 확실히 알고 있다. | 1 | 2 | 3 | 4 | 5 |
| 11. 나는 근거기반실무는 **시간이 너무 많이 소요된다**고 생각한다. | 1 | 2 | 3 | 4 | 5 |
| 12. 나는 근거기반실무를 수행하는데 **필요한 최상의 자원에 접근할 수 있다**고 믿는다. | 1 | 2 | 3 | 4 | 5 |
| 13. 나는 **근거기반실무가 어렵다**고 생각한다. | 1 | 2 | 3 | 4 | 5 |
| 14. 나는 실무에 **변화를 일으킬 만큼 충분한 근거기반실무 수행 방법을 알고 있다**. | 1 | 2 | 3 | 4 | 5 |
| 15. 나는 내가 일하는 곳에서 **근거기반실무를 적용할 수 있는 능력**이 나에게 충분히 있다고 확신한다. | 1 | 2 | 3 | 4 | 5 |
| 16. 나는 **내가 제공하는 간호가 근거에 기반하고 있다**고 믿는다. | 1 | 2 | 3 | 4 | 5 |

※ 다음은 근거기반실무에 대한 지식과 기술을 묻는 질문입니다. 정답이나 오답은 없습니다. 각 문항을 읽고 귀하가 현재 어느 정도를 갖추고 있다고 생각하는지, 가장 가까운 곳에 V 표시해주세요.

| 문 항 | 매우 부족 | ⟵ | | ⟶ | | | 매우 탁월 |
|---|---|---|---|---|---|---|---|
| 1. **간호연구 수행 능력** | 1 | 2 | 3 | 4 | 5 | 6 | 7 |
| 2. 환자 간호/치료와 관련된 근거를 찾기 위한 **IT (정보검색 및 사용) 활용 능력** | 1 | 2 | 3 | 4 | 5 | 6 | 7 |
| 3. 자신 또는 동료의 **간호실무에 대한 검토와 모니터링** | 1 | 2 | 3 | 4 | 5 | 6 | 7 |
| 4. 필요로 하는 실무지식을 **연구문제로 전환**하는 능력 | 1 | 2 | 3 | 4 | 5 | 6 | 7 |
| 5. 환자 간호/치료 관련 과학적 **지식 및 주요 정보의 종류와 출처를 알 수 있는 능력** | 1 | 2 | 3 | 4 | 5 | 6 | 7 |
| 6. 지신의 실무영역에서 **과학적 근거가 필요한 부분을 파악할 수 있는 능력** | 1 | 2 | 3 | 4 | 5 | 6 | 7 |
| 7. 실무를 위한 **근거자료를 어떻게 찾는지에 대한 지식** | 1 | 2 | 3 | 4 | 5 | 6 | 7 |
| 8. 기존의 표준에 반대되는 **근거를 비판적으로 분석하는 능력** | 1 | 2 | 3 | 4 | 5 | 6 | 7 |
| 9. 본인이 찾은 **지식/근거가 얼마나 과학적으로 타당한지를 판단할 수 있는 능력** | 1 | 2 | 3 | 4 | 5 | 6 | 7 |
| 10. 근거 자료가 **임상적으로 적용가능한**지 결정할 수 있는 능력 | 1 | 2 | 3 | 4 | 5 | 6 | 7 |
| 11. 환자의 선호도 및 병동의 **상황을 고려하여 근거(정보)를 적용할 수 있는 능력** | 1 | 2 | 3 | 4 | 5 | 6 | 7 |
| 12. 동료들과 **근거에 대한 아이디어와 정보를 공유하는 것** | 1 | 2 | 3 | 4 | 5 | 6 | 7 |
| 13. 동료에게 간호 관련 문제 및 이와 관련된 **새로운 아이디어를 전달하는 것** | 1 | 2 | 3 | 4 | 5 | 6 | 7 |
| 14. 자신이 수행한 **간호 업무와 그 결과를 평가할 수 있는 능력** | 1 | 2 | 3 | 4 | 5 | 6 | 7 |

※ 다음은 근거기반실무에 대한 조직의 준비수준에 관한 질문입니다. 정답이나 오답은 없습니다. 각 문항을 읽고 귀하가 근무하고 계시는 병원의 근거기반실무 확산을 위한 준비가 어느 정도인지 가장 적절한 곳에 V 표시해주세요.

| 문 항 | 전혀 없음 | 약간 그러함 | 다소/ 보통임 | 상당히 있음 | 매우 잘 되어(하고/ 많이) 있음 |
|---|---|---|---|---|---|
| 1. 조선대병원의 **미션과 철학**에는 근거기반실무가 명확하게 기술되어 있습니까? | 1 | 2 | 3 | 4 | 5 |
| 2. 조선대병원에서는 근거기반실무가 **얼마나 수행**되고 있다고 여깁니까? | 1 | 2 | 3 | 4 | 5 |
| 3. 조선대병원에의 **간호사**들은 근거기반실무에 어느 정도 참여하고 있습니까? | 1 | 2 | 3 | 4 | 5 |
| 4. 당신과 함께 일하고 있는 **의료팀**은 근거기반실무에 어느 정도 참여하고 있습니까? | 1 | 2 | 3 | 4 | 5 |
| 5. 조선대병원의 **행정가(경영진, 행정직)**들은 근거기반실무에 어느 정도 참여하고 있습니까?<br>(즉, 근거기반실무 착수를 위한 자원[예, 시간]과 지원을 계획하는 일) | 1 | 2 | 3 | 4 | 5 |
| 6. 조선대병원에는 근**거기반실무에 대한 지식과 기술이 매우 뛰어난 간호사**들이 충분히 확보되어 있습니까? | 1 | 2 | 3 | 4 | 5 |
| 7. 조선대병원에는 근거가 없을 때 **근거를 도출할 수 있는 간호학자들**(박사급 수준의 연구원, 박사학위 소지 간호사)들이 얼마나 있습니까? | 1 | 2 | 3 | 4 | 5 |
| 8. 조선대병원에는 일반간호사 뿐만 아니라 다른 전문간호사들에게 근거기반실무를 가르쳐 줄 수 있는 **멘토 역할을 하는 간호사**가 얼마나 있습니까? | 1 | 2 | 3 | 4 | 5 |
| 9. 조선대병원의 **의사**들은 임상 현장에서 근거기반실무를 얼마나 적용합니까? | 1 | 2 | 3 | 4 | 5 |
| 10. 조선대병원의 일반간호사들은 최상의 근거를 찾기 위하여 **질 좋은 컴퓨터와 전자 데이터베이스**를 얼마나 접하고 있습니까? | 1 | 2 | 3 | 4 | 5 |
| 11. 조선대병원의 일반간호사들은 **컴퓨터 활용 기술**이 얼마나 능숙합니까? | 1 | 2 | 3 | 4 | 5 |
| 12. 조선대병원에서 이용할 수 있는 **도서관 사서(대학 중앙도서관 사서 포함)는 근거기반실무에 대한 지식과 기술**을 얼마나 갖고 있습니까? | 1 | 2 | 3 | 4 | 5 |
| 13. 조선대병원에서 이용할 수 있는 **도서관 사서(대학 중앙도서관 사서 포함)가 근거 검색에 얼마나 활용되나요?** | 1 | 2 | 3 | 4 | 5 |
| 14. 조선대병원에는 근**거기반실무를 지원하는 자원**은 얼마나 있습니까?<br>(근거기반실무 세미나/워크숍 교육, 인사고과 반영, 수당, 컴퓨터, 멘토, 도서관, 도서관 사서 등) | 1 | 2 | 3 | 4 | 5 |

| 문 항 | 전혀 없음 | 거의 없음 | 보통 있음 | 대체로 있음 | 매우 잘 되어(하고/많이) 있음 |
|---|---|---|---|---|---|
| * 조선대병원에는 다음의 각 영역에서, **근거기반실무 챔피언** (전문가 혹은 근거기반실무를 추진시킬 수 있는 인력)이 있다고 생각하십니까? | | | | | |
| 15. 간호행정가(간호행정부서, 수간호사, 간호팀장, 간호부장 등) | 1 | 2 | 3 | 4 | 5 |
| 16. 의사 | 1 | 2 | 3 | 4 | 5 |
| 17. 간호교육자 | 1 | 2 | 3 | 4 | 5 |
| 18. 전문간호사 | 1 | 2 | 3 | 4 | 5 |
| 19. 일반간호사 | 1 | 2 | 3 | 4 | 5 |
| 20. 조선대병원에는 ‘성과를 측정하고 공유하는 것’이 조직 문화로 얼마나 자리 잡고 있다고 생각하십니까? | 1 | 2 | 3 | 4 | 5 |
| **문 항** | | | | | |
| * **조선대병원의 주요 정책 관련 의사결정**에 있어서 다음의 각 그룹이 참여하는 정도는? | | | | | |
| 21. 직접 간호 제공자 (간호사) | 0% | 25% | 50% | 75% | 100% |
| 22. 병원 경영진 | 0% | 25% | 50% | 75% | 100% |
| 23. 의사나 타 건강관리 제공자 그룹 | 0% | 25% | 50% | 75% | 100% |
| **문 항** | | | | | |
| 24. 종합적으로, **조선대학교병원의 근거기반실무 준비도**를 어떻게 평가하십니까? | 전혀 준비 안 됨 ① | 준비 중임 ② | 준비완료 /미수행 ③ | 막 수행 시작 ④ | 수행 잘됨 ⑤ |
| 25. 6개월 전과 비교해 볼 때, 귀 병원에는 근거기반실무 문화로 나아가는 움직임이 얼마나 있습니까? (옆에 빈칸에 표시를 해주세요) | □ 전혀 없음 | □ | □ | □ | □ 매우 잘 됨 |

※ 다음은 근거기반실무의 수행에 대한 질문입니다. 얼마나 자주 해야 한다고 정해진 기준은 없습니다.
귀하께서 **지난 8주간** 아래 항목들을 얼마나 자주 시행했는지, 해당하는 곳에 V 표시해주세요.

| 문 항 | 0회 | 1-3회 | 4-5회 | 6-7회 | 8회 이상 |
|---|---|---|---|---|---|
| 1. 나는 임상실무를 변화시키기 위해 근거를 활용하였다. | 0회 | 1-3회 | 4-5회 | 6-7회 | 8회 이상 |
| 2. 나는 연구에서 나온 근거를 비판적으로 평가하였다. | 0회 | 1-3회 | 4-5회 | 6-7회 | 8회 이상 |
| 3. 나는 나의 임상실무에 대한 PICO* 형태의 임상질문을 도출하였다.<br>*P: 환자, 관심 집단, 간호문제, I: 환자를 위한 새로운 중재나 간호방법, C: 기존의 간호중재나 비교할 중재, O: 기대하는 결과 | 0회 | 1-3회 | 4-5회 | 6-7회 | 8회 이상 |
| 4. 나는 연구에서 나온 근거에 대해 동료와 비공식적으로 토론하였다. | 0회 | 1-3회 | 4-5회 | 6-7회 | 8회 이상 |
| 5. 나는 환자 문제에 대한 자료를 별도로 수집하였다. | 0회 | 1-3회 | 4-5회 | 6-7회 | 8회 이상 |
| 6. 나는 연구에서 나온 근거를 보고서나 프리젠테이션 형태로 2명 이상의 동료와 공유하였다. | 0회 | 1-3회 | 4-5회 | 6-7회 | 8회 이상 |
| 7. 나는 임상실무의 변화에 따른 결과를 평가하였다. | 0회 | 1-3회 | 4-5회 | 6-7회 | 8회 이상 |
| 8. 나는 근거기반 실무지침을 동료와 공유하였다. | 0회 | 1-3회 | 4-5회 | 6-7회 | 8회 이상 |
| 9. 나는 연구에서 나온 근거를 환자나 그 가족과 공유하였다. | 0회 | 1-3회 | 4-5회 | 6-7회 | 8회 이상 |
| 10. 나는 연구에서 나온 근거를 다학제적 팀원들(의사, 전문간호사, 영양사, 약사 등)과 공유하였다. | 0회 | 1-3회 | 4-5회 | 6-7회 | 8회 이상 |
| 11. 나는 임상 연구를 읽고 비판적으로 평가하였다. | 0회 | 1-3회 | 4-5회 | 6-7회 | 8회 이상 |
| 12. 나는 체계적 고찰에 대한 자료를 보기 위해 코크란(Cochrane)에 접속하였다. | 0회 | 1-3회 | 4-5회 | 6-7회 | 8회 이상 |
| 13. 나는 임상지침데이터베이스 National Guideline Clearinghouse(NGC)*에 접속하였다.<br>*미국 정부에서 주도하는 임상지침 데이터베이스로서 개발된 진료지침, 진료지침의 개발 방법론, 활용방안을 웹을 통해 제공함. | 0회 | 1-3회 | 4-5회 | 6-7회 | 8회 이상 |
| 14. 나는 내가 일하는 곳의 임상실무를 변화시키기 위해 근거기반실무지침이나 체계적 고찰을 이용하였다. | 0회 | 1-3회 | 4-5회 | 6-7회 | 8회 이상 |
| 15. 나는 환자 결과 자료를 수집하여 간호 계획을 얼마나 달성했는지 평가하였다. | 0회 | 1-3회 | 4-5회 | 6-7회 | 8회 이상 |
| 16. 나는 수집한 환자 결과 자료를 동료들과 공유하였다. | 0회 | 1-3회 | 4-5회 | 6-7회 | 8회 이상 |
| 17. 나는 환자의 결과 자료에 근거하여 실무를 변화시켰다. | 0회 | 1-3회 | 4-5회 | 6-7회 | 8회 이상 |
| 18. 나는 동료들에게 근거기반실무를 적용하도록 권장하였다. | 0회 | 1-3회 | 4-5회 | 6-7회 | 8회 이상 |

※ 다음은 일반적 특성에 관한 내용입니다. 해당란에 V 표시하거나 직접 기록해주세요.

| | |
|---|---|
| 성별 | ☐ 여 ☐ 남 |
| 연령 | 만 ________ 세 |
| 교육정도 | ☐ 전문대졸 ☐ 학사 ☐ 석사과정 중 혹은 수료 ☐ 석사 졸업<br>☐ 박사과정 중 혹은 수료 ☐ 박사 졸업 ☐ 기타 ______________ |
| 최종학위 취득년도 (졸업년도) | ________ 년 |
| 직책 | ☐ 일반간호사 ☐ 주임(책임, charge)간호사 ☐ 전문/전담간호사<br>☐ 수간호사 ☐ 간호행정관리자 (간호팀장 이상) ☐ 기타 ______________ |
| 근무경력 | 총 임상경력: ______ 년 ______ 개월 / 현재 부서에서의 근무경력: _____ 년 ____ 개월 |
| 현 근무병동 | ☐ 병동 (_____병동) ☐ 외래 ☐ 외래특수(검사)파트<br>☐ 중환자실 ☐ 응급실 ☐ 분만실<br>☐ 수술실 ☐ 마취회복실 ☐ 인공신장실<br>☐ 간호부 행정부서 ☐ 기타 ______________ |

| | | |
|---|---|---|
| 귀하는 '**간호연구, 연구방법론**'에 관한 강좌를 이수한 적이 있습니까? | ☐ 예 (학부____/대학원____/병원____) | ☐ 아니오 |
| 귀하는 '**통계학, 간호통계와 실습**'에 관한 강좌를 이수한 적이 있습니까? | ☐ 예 (학부____/대학원____/병원____) | ☐ 아니오 |
| 귀하는 '**근거기반실무**'에 관한 강좌를 이수한 적이 있습니까? | ☐ 예 (학부____/대학원____/병원____) | ☐ 아니오 |
| 귀하는 **연구에 직접 혹은 간접적으로 참여한 경험**이 있습니까? | ☐ 예 (학부____/대학원____/병원____) | ☐ 아니오 |
| 귀하는 **학회나 학술단체에 소속**되어 있습니까? | ☐ 예 (단체명: ___________________) | ☐ 아니오 |
| 귀하는 정기적으로 **학술대회에 참석**하십니까? | ☐ 예 (작년 기준 연간 횟수: ______회) | ☐ 아니오 |
| 귀하는 '근거기반실무'라는 **용어가 친숙**합니까? | ☐ 예 | ☐ 아니오 |
